# Estimating the causal effect of a quality assurance program on quality of care in Germany

**DOI:** 10.1186/s12913-025-12939-8

**Published:** 2025-06-09

**Authors:** Stefan Gehrig, Britta Zander-Jentsch, Maurilio Gutzeit, Silvia Klein, Johannes Rauh

**Affiliations:** 1 Institute for Quality Assurance and Transparency in Healthcare (IQTIG), Katharina-Heinroth-Ufer 1, Berlin, 10787 Germany; 2 IU International University of Applied Sciences, Juri-Gagarin-Ring 152, Erfurt, 99084 Germany

**Keywords:** Quality of care, Quality indicators, Quality policy, Hospital capacity planning, Policy evaluation, Difference-in-differences

## Abstract

**Background:**

Inpatient safety can benefit from effective policies for hospital quality assurance. Following legal reform to put quality of care more center stage in hospital capacity planning, a new quality assurance program was introduced in Germany in 2017. The program was based on pre-existing quality indicators and included new policy design components emphasizing hospital accountability. Learning if the policy was effective is important, but challenging in the observational setting.

**Methods:**

We adapt a quasi-experimental difference-in-differences approach to estimate the causal effect of the program on quality of care in the 4 years after program onset. A control group of indicators from other clinical areas is carefully selected to compare trends in process quality.

**Results:**

Results show a relevant reduction in risk of adverse care events nationally in the affected patient population relative to a counterfactual scenario of no program adoption. The effect emerged over the first two program years before plateauing.

**Conclusion:**

The study allows to learn about important design components in healthcare quality assurance. Among our discussed mechanisms for the documented improvement in process quality are the initial announcements of legal consequences and high public attention. These led hospitals to initiate quality improvement, to seek dialogue with state agencies, and to put more efforts into submitting correct quality data. Methodologically, we show how to adapt rigorous study designs to quality assurance evaluation, considering also their assumptions and limitations.

**Supplementary Information:**

The online version contains supplementary material available at 10.1186/s12913-025-12939-8.

## Background

Inpatient safety can benefit from effective policies for hospital quality assurance. Yet, design elements and implementations of policies aiming to improve quality of care delivery vary widely, with often only limited evidence of their effectiveness [1–3]. In Germany, a core element of Hospital Structure Act from 2015 was the introduction of a program using quality indicators for hospital capacity planning [4]. This was intended to assign quality of care a central role in hospital planning decisions of regulators. Hospitals that would significantly and enduringly demonstrate inadequate quality should be completely or partially removed from public reimbursement plans. For Germany, this development constituted a major shift from the use of healthcare quality data primarily for quality promotion and public information towards increased accountability [5].

As a result, in 2017, the Federal Joint Committee (G-BA [Gemeinsamer Bundesausschuss]) adopted a new directive and corresponding quality assurance program to comply with legislation. The directive specifies the processes of data collection, data validation, statistical and subsequent qualitative assessment of hospitals’ results, as well as public reporting for all licensed hospitals in Germany [6].

Based on recommendations by the Federal Institute for Quality Assurance and Transparency in Healthcare (IQTIG [Institut für Qualitätssicherung und Transparenz im Gesundheitswesen]), the G-BA determined a set of 11 already pre-existing quality indicators from the clinical areas of gynecological surgery, obstetrics and breast surgery to be monitored as part of the new program [7]. The selected indicators were assumed to reflect critical aspects of patient safety and to constitute legitimate quality requirements (see [5] for more details on the development and political context of the program). The directive specified a bundle of procedural modifications for the 11 indicators, including

- time-constant indicator reference ranges and a statistical method to infer whether a hospital’s performance was consistent with that range,
- a comprehensive data validation process to scrutinize documented data,
- a centralized and standardized “commenting procedure” requiring that hospitals explain outlying results,
- as well as faster, more-easily comparable public reporting of indicator results.

These changes were supposed to facilitate the detection of healthcare services that were of so-called *insufficient quality* [8]. By setting standards in the form of quantitative reference ranges for measurable quality dimensions, combining them with financial/regulatory consequences, and putting a central, transparent reporting system in place, the program exhibits some typical policy ingredients for hospital quality accountability [3, 9]. Indeed, the program raised the visibility and importance of the 11 hospital quality indicators in the eyes of policy-makers and the public. National press coverage was high after the first release of online reports of hospital results (e.g., [10]).

A remaining key policy question is, whether – and, potentially, how – the program has been able to improve inpatient quality of care in Germany. Initial research by [5] suggests that the program has led to better overall awareness of quality aspects especially among providers and regulators, and has revealed the importance of data validation. Time series of national-level quality indicator results and of the number of outlying hospitals demonstrate that documented quality of care has improved for the 11 included indicators since introduction of the program. Therefore, the mere longitudinal trend suggests a positive influence of the program.

However, positive time trends in the quality of medical procedures are generally to be expected and do not yet point to an effect caused by the program itself. For example, technological progress, increased healthcare spending, or new evidence-based clinical practices might improve processes and outcomes over time, even in the absence of novel policies that target quality assurance. Moreover, already before the onset of the program, the indicators’ results had been reported regularly as part of statutory quality assurance in Germany. Such mandatory public reporting alone could have positive effects on long-term quality improvements [11]. In fact, to date, there is no evidence the program affected regulators’ hospital planning decisions [5]. It is unclear how quality of care in the 11 indicators would have developed without the new program. This reflects the fundamental causal inference problem of health policy impact evaluation from observational (non-experimental) data [12, 13]. The challenge is particularly acute in the realm of quality policies: In Germany, effectiveness of healthcare quality assurance has rarely been evaluated rigorously [2].

In this paper, we adopt a quasi-experimental study design for identifying a causal effect of the program. Tailoring a classic difference-in-differences (DiD) approach to the current setting, we attempt to account for general time trends in quality of care that are unrelated to the program. The principal approach is to select as a comparison group other quality indicators. That is, to observe German patients admitted in the same years, but in other clinical areas and for other medical conditions, whose quality of care is measured by other indicators. Under a couple of assumptions, the plausibility of which we will discuss, this allows to infer whether the introduction of the program had a causal impact on the quality of care in the clinical areas it covered, and to estimate the magnitude of this causal impact.

Our research question is relevant beyond the scope of the specific program under study here. Given the heterogeneity of healthcare and quality assurance systems across countries, it is important to understand in which contexts which policy levers work (better than others). Previous research has compared approaches of quality policy between countries [9, 14, 15]. This study takes a different angle, more similar to the work of [11]. We investigate variation in regulatory regimes between different sets of quality indicators over the same time frame and within the same country – and hence, by majority within the same hospitals. Specifically, we want to examine whether policy decisions towards increased accountability and attention to quality in selected clinical areas can improve quality of care. This sheds light on the question whether similar programs or design components should be prioritized nationally and internationally.

## Methods

### Data

#### Data source

We draw on published quality indicator results for the observation period from 2013 (4 years before program introduction) to 2020 (4 years after program introduction). Results are annually reported to the public by IQTIG as part of the statutory quality assurance in Germany for hundreds of quality indicators spanning various clinical areas. The routine collection of the underlying case-level data for quality assurance is mandatory for healthcare providers. Results include the 11 indicators selected for the program. Public access to the national-level results reports and all indicator definitions is provided at https://iqtig.org/. Historical data not available on the website can be requested from IQTIG.^1^

Since the start of the program, both results *before* and *after* external data validation have been annually reported for the 11 program indicators. The latter includes potential recalculations of indicator results based on corrected data entries. Whereas some data validation procedures had already existed before in German statutory quality assurance [16], results recalculation was a novelty of the program. In this study, we deliberately only use results *before* data validation for all analyses. First, this ensures a constant level of scrutiny for data from before and after program implementation. Second, this improves comparability to indicators outside the program, for which neither data validation nor recalculation took place.^2^

#### Quality indicators

##### General background

Quality indicators are designed to quantitatively measure different quality aspects of healthcare delivery [17]. Each indicator in German statutory healthcare quality assurance comes with a precise definition of patient population, medical procedure and event of interest, following evidence-based recommendations. Most indicators are defined as “number of events of interest divided by number of cases”, with results reported as proportions. Events of interest could be adverse outcomes like mortality, but also poor processes like delayed diagnostic procedures after admission, or guideline non-conforming indications. Such events are henceforth collectively called *adverse care events*, irrespective of whether they reflect shortcomings in outcome, process or indication quality. Every quality indicator is assigned a reference range against which providers are benchmarked. The reference range is either fixed or based on a percentile of the distribution of results of all hospitals. For legal certainty, reference ranges for the 11 indicators in the program had to be defined and published prospectively [7]. Therefore, these reference ranges have been fixed and constant since 2017. Other indicators not in the program might be assigned percentile-based reference ranges, which by definition can only be applied when the data is observed, or fixed reference ranges that are occasionally altered. Some quality indicators are risk adjusted, typically outcome indicators defined in terms of standardized morbidity ratios [18].

##### Selection of a control group

In 2016, pre-existing quality indicators were selected to be included in the program in two steps. First, only clinical areas with good quality indicator coverage were considered [7]. Second, indicators from these areas had to pass five formal criteria to be recommended for the program [7]. These were (A) relevance for preventing severe patient hazards, (B) operational maturity, (C) adequate risk adjustment (if necessary), (D) evidence for the legitimacy of the quality requirement, and (E) no other reasons that speak against their selection.

For our study design based on DiD, we need both a *program group* and a *control group* of quality indicators. Note that this differs from standard applications of DiD, where groups to be compared would rather consist of, for example, states, regions or hospitals. We explain our adaptation of the DiD approach and its assumptions in more detail in the “Empirical strategy and estimation” section.

It is important for the validity of the design that the two quality indicator groups are well comparable (see “Empirical strategy and estimation” section and Supplementary information A). Thus, a potential control group of indicators is selected in a way that broadly mirrors the original selection of program indicators along above criteria, but for other clinical areas whose indicators were not already reviewed for program inclusion in the original selection procedure. Using the complete database of IQTIG’s hospital quality indicators as starting point, we apply the following requirements in an automated selection procedure to arrive at candidate indicators for the control group:

1. The clinical area was not already selected for possible program inclusion by [7]. This removes indicators from the areas gynecological surgery, obstetrics, breast surgery (all of which became part of the program) and heart surgery (whose indicators did not pass the five eligibility criteria).
2. In 2014 and 2015, the two years prior to program design, indicators’ definitions were stable and they were fully in operation. Specifically, (i) indicator definitions were as least “moderately comparable” in each year^3^, (ii) indicators had a reference range in place, and (iii) indicators were deemed sufficiently valid by expert panels for an assessment of national need for action (all reflecting criterion B from above).
3. Indicators are risk adjusted if they measure patient outcomes (reflecting criterion C from above).

Although this closely reproduces above-mentioned criteria B and C from [7], the other criteria A, D and E would require indicator-specific in-depth reviews, expert discussions and literature research. This was not feasible as part of this study.

##### Final analysis data set

The study design depends on longitudinal comparability of quality indicator results over 8 years (see “Empirical strategy and estimation” section). Therefore, the program group of 11 indicators and all candidate indicators for the control group were further narrowed down. Quality indicators were only included in analysis if they additionally satisfied the following two criteria.

1. *They have been in place as quality indicator at least for the whole observation period from 2013 to 2020 and have not been strongly modified between years.* The latter was defined such that from 2014 on, they were always at least “moderately comparable” to the previous year’s result. Program indicators, for which major modifications were strictly discouraged for legal reasons, were all retained by default in this step.^4^
2. *They measure process or indication quality.* IQTIG classifies indicators into measuring indication, process or outcome quality. Time series of outcome indicators (e.g., mortality, surgical complications, patient mobility) over longer periods can be difficult to interpret, for several reasons. First, if they are not adjusted for patient risk factors, they might be heavily affected by trends in patient population characteristics. For example, [5] explicitly note that changes in the unadjusted proportion of adverse events for one of the two risk-adjusted indicators in the program (measuring injuries during gynecological surgery) “may be explained by demographic changes in the patient population over the course of several years.” Second, even if they are adjusted for some observed risk factors, it is possible that changes in unobserved factors affect the time trend. Third, statistical risk adjustment models used by IQTIG are updated regularly, typically on an annual basis. Consequently, which and how patient characteristics are adjusted for varies across years, along with the “reference population” used for standardization (see [18]). This undermines comparisons of nationwide risk-adjusted results from different annual reports. Developing a new and time-constant risk adjustment model for the purpose of this study was not possible due to the lack of patient-level data. In sum, these issues present a serious risk of bias: As soon as different indicators are differently affected by year-to-year changes in unaccounted risk factors, this could induce confounding for our DiD approach [19]. Therefore, outcome indicators were removed from analysis. In what follows, we use the term process quality to cover both process and indication quality, as is common the field (e.g., [20]).

This leads to the program and control group consisting of 9 and 10 indicators, respectively. Hence, in total, the data set contains $\:\left(9+10\right)\times\:8=152$ annual national-level results. All quality indicators included in the study are summarized in Table 1, along with aggregate counts of their population size and adverse care events. They cover more than 12 million patient observations. For indicators which originally count favorable rather than adverse care events the coding of events was reversed. Time series over the whole observation period for all indicators are presented in Supplementary information B.

**Table 1:** Description of quality indicators included in the study in program and control group

ID	Clinical area	Description	Population size (2013–2020)	Adverse care events (2013–2020)	Indicator type
Program					
2163	Breast surgery	Primary axillary dissection in DCIS	54,287	101 (0.19%)	Indication
52279	Breast surgery	Intraoperative specimen sonography or x-ray with sonographic wire marking (reverse coded)	188,749	18,246 (9.67%)	Process
52330	Breast surgery	Intraoperative specimen sonography or x-ray with mammographic wire marking (reverse coded)	157,010	2,273 (1.45%)	Process
10211	Gynecological surgery	Complete removal of the ovary or adnexa without pathological findings	130,503	13,694 (10.49%)	Indication
12874	Gynecological surgery	Missing histology after isolated ovarian surgery with tissue removal	301,726	3,927 (1.30%)	Indication
1058	Obstetrics	D-D time in emergency cesarean section > 20 min	76,475	340 (0.44%)	Process
318	Obstetrics	Presence of a paediatrician at premature births (reverse coded)	203,198	7,253 (3.57%)	Process
330	Obstetrics	Antenatal corticosteroid therapy in premature births with prepartum hospitalization for at least two calendar days (reverse coded)	61,027	2,048 (3.36%)	Process
50045	Obstetrics	Perioperative antibiotic prophylaxis in cesarean section delivery (reverse coded)	1,827,564	25,805 (1.41%)	Process
Control					
51437	Carotid artery revascularization	Indication in asymptomatic carotid artery stenosis - catheter-supported (reverse coded)	24,242	630 (2.60%)	Indication
51443	Carotid artery revascularization	Indication in symptomatic carotid artery stenosis - catheter-supported (reverse coded)	15,261	152 (1.00%)	Indication
603	Carotid artery revascularization	Indication in asymptomatic carotid artery stenosis - open surgery (reverse coded)	113,245	1,669 (1.47%)	Indication
604	Carotid artery revascularization	Indication in symptomatic carotid artery stenosis - open surgery (reverse coded)	72,008	393 (0.55%)	Indication
2005	Community-acquired pneumonia	First blood gas analysis or pulse oximetry within 8 h after admission (reverse coded)	2,182,966	38,560 (1.77%)	Process
2009	Community-acquired pneumonia	Antimicrobial therapy within 8 h after hospitalization (reverse coded)	1,774,816	87,175 (4.91%)	Process
2013	Community-acquired pneumonia	Early mobilization within 24 h after admission for risk class 1 (CRB-65-SCORE = 1 or 2) (reverse coded)	1,047,287	71,615 (6.84%)	Process
2028	Community-acquired pneumonia	Completely measured clinical stability criteria at discharge (reverse coded)	1,387,236	65,062 (4.69%)	Process
50722	Community-acquired pneumonia	Determination of respiratory rate on admission (reverse coded)	2,136,942	88,926 (4.16%)	Process
50063	Neonatology	Hearing test performed (reverse coded)	759,748	20,548 (2.70%)	Process

### Empirical strategy and estimation

We use a DiD approach to estimate the causal effect of program introduction on adverse care events. DiD is a widely used quasi-experimental method for impact evaluation of health policies [12, 13, 21]. It is well-suited for the retrospective analysis of observational, routinely collected healthcare data (see [22] for an example from Germany).

In the current study, a DiD design with the same quality indicators in facilities in the program and a control group of facilities is not possible. The program was introduced simultaneously for all hospitals in Germany. Such lack of regional variation in intervention status or timing has been cited as a common challenge when evaluating quality impacts of national policies [23]. To nevertheless extend the mere before-after comparison by [5], we exploit that some quality indicators fell under regulation by the program starting 2017, while others did not. This creates the opportunity to define a program group and control group of indicators. The design is not fully novel. For example, it is similar in spirit to the “controlled before–after analysis” of quality indicators by [11].^5^

Nevertheless, the approach deviates from other DiD designs in the literature on quality policy impacts. More conventionally, DiD compares the same outcome measure among patients in treated and untreated units, which could be states, regions or hospitals. Examples of such DiD studies are evaluations of the healthcare quality impact of performance-based incentives from Africa [24, 25], or of a ministry-led quality reporting initiative from Japan [26]. Instead, we are comparing patients admitted to the same hospitals, but in other clinical areas and for other medical treatments. The outcome variable comprises different types of care events across indicators (Table 1). The important joint characteristic of patients in both groups is that their quality of care delivery is measured by established indicators. Hence, our treated units are quality indicators (i.e., the patient population, medical procedure and event they are defined to capture). The data are annually repeated cross-sections of all national patients who fall into the target population of an indicator selected for analysis (see “Quality indicators” section). In summary, at the heart of our adapted DiD approach is a comparison of changes in the average proportion of adverse care events in the pre- and post-program period between program indicators and a group of comparable quality indicators, for which the program was never introduced.

The adapted DiD design comes with special challenges. First, even though DiD accounts for time-constant differences in adverse care event proportion between groups, there is still risk of confounding by quality indicator. The relative shares of patient populations of the various indicators within a group are not perfectly constant over time. As indicators refer to vastly different populations and care processes, they also differ in the risk of adverse care events – sometimes by orders of magnitude (see Table 1). Hence, indicator could be a confounding variable even in a DiD design due to its correlation with the outcome variable combined with its potentially time-varying distribution [19]. Importantly, our estimation strategy adjusts for such effects by giving each indicator constant weight over the years (and, in fact, equal weight, as we discuss in Supplementary information A). Second, the design puts additional burden on the *no interference* assumption required for causal inference (Supplementary information A). As patients affected and not affected by the policy are treated in the same hospitals, there could be spillovers. Yet, the assumption is still largely plausible. For example, the patient populations in the two groups have no overlap with respect to the clinical areas of their medical conditions (see Table 1). We briefly return to this question when discussing limitations in the “Potential limitations” section.

**Fig. 1:**
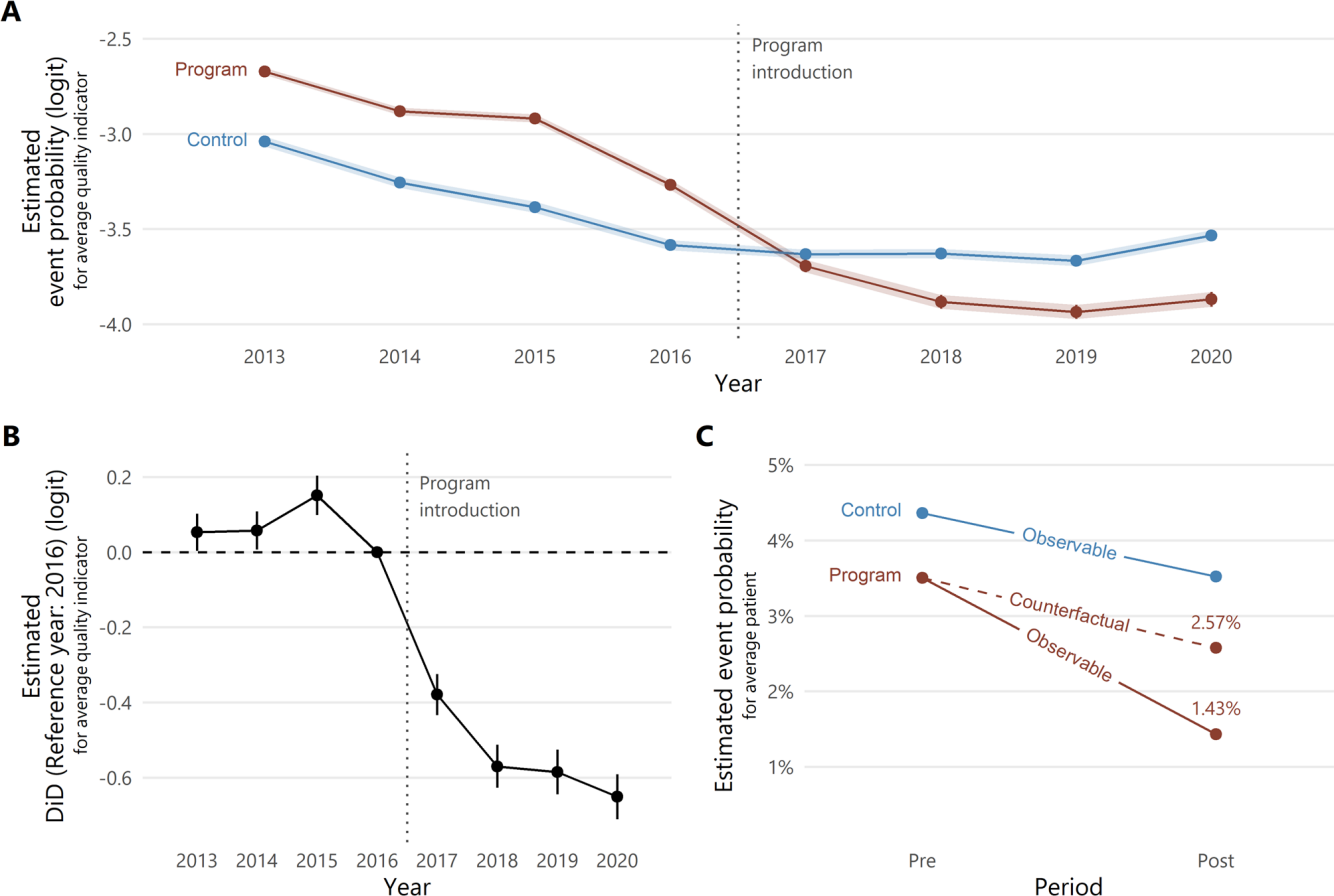
**A** Estimated average pre- and post-trends of program and control group of quality indicators on the logit scale, with 95% confidence intervals. **B** Event study plot showing DiD estimates for each year relative to the difference between quality indicator groups in the year 2016, with 95% confidence intervals. **C** Point estimates of observable and counterfactual average probability of an adverse care event in both periods and groups

As always in DiD, the most critical and not directly testable assumption are *parallel counterfactual trends* (e.g., [27]). It states that in the absence of the program, the results for patients in the program group would have developed in parallel to the those in the control group. More precisely, the following assumption is made: Without the program, the average indicator result in the program group would have developed in parallel to the average indicator result in the control group on the logit scale. By using a DiD on the logit transformation for the means, the DiD design accommodates binary outcome variables, following previous work in economics [28, 29]. We argue for the assumption’s plausibility in more detail in Supplementary information A. Note that the 11 indicators for the program were selected according to clear and well-documented criteria [7], and subsequently adopted by all German hospitals. This is a strength of our adapted DiD design: In contrast to many other DiD designs with intervention assignment on the hospital level, there was no voluntary component in adopting the program. No hidden factors/trends associated with actively opting into the policy can endanger the parallel trends assumption. Indeed, lending credibility to the assumption, average indicator results move approximately in parallel between the two groups prior to the program, with a clear diverging trend only after program onset (Fig. 1). Under our assumptions, we can interpret estimated deviations from the parallel trend as estimate of the causal program effect. The effect is transformed into a causal risk ratio that marginalizes over the patients (specifically, the years and indicators they were treated in) who were actually affected by the program after its introduction. Thus, this risk ratio is a measure of the average treatment effect on the treated (ATT). Supplementary information A in detail defines all quantities of interest and their estimation, along with further discussion of methods and assumptions.

## Results

The estimate of the DiD between pre- and post-program period on the logit scale, resulting from averaging over quality indicators, is -0.61 (95% CI: -0.64; -0.58). This implies an estimated odds ratio of $\:\text{exp}\left(-0.61\right)=0.54$. That is, under our assumptions, the odds of an adverse care event *for the average program indicator* in an average year were almost halved due to the program. The effect estimate can be transformed into a marginal causal risk ratio for the ATT. It takes into account that population sizes vary between years and indicators. The resulting risk ratio estimate of 0.56 (95% CI: 0.54; 0.57) implies that the probability of a poor clinical process was reduced by 44%* for the average patient treated after program introduction* in years 2017 to 2020, relative to what would have happened without the program.

Figure 1 gives a graphical summary of the results. Figure 1A shows that average indicator results in the two groups are moving approximately in parallel before program introduction, but diverge drastically afterwards. This pattern is also reflected in the event-study plot in Fig. 1B that shows year-wise DiD estimates. In Fig. 1C, we visualize point estimates of the estimated event probability for all group-period cells for the average patient (connected by solid lines). In addition, the dashed line shows the trend we would have expected if the program had not been introduced, i.e., the counterfactual trend based on our assumptions (most importantly parallel counterfactual trends of the average quality indicator on the logit scale). The ratio of estimated counterfactual and observable probabilities shown in Fig. 1C, $$\begin{aligned} 1.43\%/2.57\%=0.56 \end{aligned}$$, is the estimate of the marginal causal risk ratio for the ATT reported above.

It is possible to transform the relative effect estimate into an absolute estimate. The total number of patients in the selected program indicators who, according to quality documentation, received adequate care because of the program (i.e., for whom deviations from evidence-based processes were avoided due to the program) in the years 2017 to 2020 is approximately 17,300 (95% CI: 16,400; 18,300). Benefit in absolute numbers is larger for indicators with larger populations and more frequent adverse care events. With its large volume of procedures (more than 200,000 per year), most process deviations were prevented in the indicator on antibiotic prophylaxis in cesarean section delivery (ID 50054, Table 1), adding up to 7,200 (95% CI: 6,800; 7,700) over the 4 post-program years.

The above main result was obtained by aggregating over all pre- and post-program years, respectively. However, Fig. 1B indicates that the program effect, shown here as DiD for the average indicator on the logit scale, is dynamic. After a rapid and strong increase in effect in the first two years, the effect size plateaus from 2018 onwards, with a slight increase again in 2020.

## Discussion

### Mechanisms

The results suggest that the directive on the use of quality indicators for hospital capacity planning and the associated program had a positive causal effect on process quality of inpatient care in Germany – and in particular so during the initial years. Seven years have passed since the first indicator results from the program were published in 2018 and an in-depth, qualitative evaluation of program implementation and achievement of its objectives has been conducted [30]. This allows a broader contextualization of our quantitative results and informed discussion of effect mechanisms.

The effect cannot be explained by a program-induced shift in which hospitals provide the medical procedures.^6^ No hospitals or departments were shut down due to poor program indicator results, contrary to the intention of the legal reform. Consequences of the program on hospital capacity planning decisions by federal states were generally small (reasons are discussed in more detail by [30]). We are also not aware of developments in the German healthcare system during the observation period that could have led to rapid, large-scale changes of hospitals’ market shares in the studied medical procedures. Looking at the number of providers in the analyzed indicators, no major discontinuities appear at program onset (Supplementary information B). The long-term trend of declining hospital numbers in Germany can be recognized for some of them. Therefore, rather than by reallocating patients, the effect is likely at least partially explained by how the program changed expectations among practicing providers. Its introduction sent a strong signal about the importance of quality of care in selected indicators – a signal whose credibility might have deteriorated over years of lacking legal consequences, shrinking public attention and rising concerns that the indicator set might not adequately reflect quality of entire clinical departments. This could have led the effect size to eventually plateau.

There is complementary evidence that the program triggered steps towards quality improvements by hospitals [30]. For example, multiple federal states used program indicator results to initiate dialogues with hospitals on quality improvement measures. Some hospitals took their own measures, e.g., by exchanging their affiliated staff with permanent staff or by more strongly emphasizing guideline compliance.

Besides that, it is plausible that the announcement of the data validation procedure led hospitals to put more effort into submitting correct quality data. Indeed, during data validation, incorrect records were often found to be to the disadvantage of providers [5]. Errors in documentation that lead to negatively biased indicator results have already been observed in earlier quality data validations in Germany [16]. Increased focus on data quality by providers could therefore also have contributed to the estimated impact. In a somewhat related example from the Netherlands, a “need to score” on a newly established policy-relevant indicator likely led hospitals to adjust their reporting behavior – in their case even to falsely enhance their results [31]. Although intentional misreporting is a possibility also in the program we study, it is made unattractive by the data validation procedure, which a (partially) random sample of hospitals had to undergo every year [7].

Why did the program effect settle at an achieved level after two years, rather than continue to grow over time (Fig. 1B)? Besides declining credibility of legal threats and loss of public attention to the program, this might be linked to ceiling effects. It has been argued that beyond a certain level of “achievable” quality of care, quality indicator results are difficult to be improved further (e.g., [32]). The estimated time series in Fig. 1A might show how the program affected the priority of providers in achieving that level: Quality was improving already prior to the program in both indicator groups at a similar pace. After the program initially led hospitals to put particularly strong effort in improving program indicator results, it is possible that potential for improvement had quickly been exhausted. In this view, the program mainly accelerated the attainment of achievable quality gains in the program indicators. Similar “boom-and-plateau” dynamics in quality of care were observed after England’s pay-for-performance scheme [33] or Germany’s public reporting mandate [11], where authors concluded that policies for quality improvements require “sufficient potential for exploitation” (p. 776).

In 2020, a decrease in process quality across both groups and widening of the gap between groups can be observed (Fig. 1A). Probably, this is due to the COVID-19 pandemic: As in other countries, routine care in German hospitals was greatly affected across all clinical areas [34]. Two process indicators for the treatment of respiratory infections were particularly negatively affected by the pandemic (IDs 2009, 2013; see their time trends in Supplementary information B). As both are in the control group, this drives the renewed increase of effect size for 2020.^7^

### Policy implications

Despite the suggested positive effect of the program on inpatient quality of care, IQTIG has recommended to not continue the program in its current design. The ambitious goal of the directive to put quality indicators more center stage in federal hospital capacity planning has arguably not materialized, possibly due to obstacles posed by current legislation [30]. A challenge for the national program was that hospital planning in Germany falls under the authority of individual states. As a consequence, no uniform application of program indicator results was established. Yet, the analysis of program impact in this study together with other in-depth evaluations [5, 30] have generated valuable insights into the effectiveness of healthcare quality policy instruments. As a consequence, program features that have proven beneficial could be integrated into existing statutory quality assurance (as also argued by [36]). They could also be used to devise new instruments against the background of Germany’s ongoing debate about quality-oriented hospital capacity planning and upcoming large-scale hospital reforms passed in 2024.

Among the most important design components of the program are probably (i) the announcement of regulatory consequences on hospital status when delivering poor quality of care, defined by a fixed reference range, (ii) the rigorous data validation and commenting procedure for outlying providers according to nationwide standardized criteria, and (iii) fast and accessible public reporting of indicator results (see also [11]). We believe that these factors in combination explain large parts of the positive results of this impact evaluation by creating hospital accountability and setting effective incentives. For example, a merely voluntary quality reporting project in Japan did not show short-term improvements in outcome and process indicators among cardiovascular patients [26]. At the same time, continuous effects probably require continuous action – both because providers might adapt when they recognize that policies have no bite, and because remaining potential for improvement might shift between indicators or medical specialties over time.

### Potential limitations

Our central assumption of parallel counterfactual trends for the average quality indicator cannot be tested and could be violated. This would invalidate the causal effect estimate from DiD. Yet, we have laid out plausible arguments that would favor the assumption (Supplementary information A). For example, unlike in many other policy evaluation settings, the assignment of the program followed well-documented criteria, none of which incorporated trends or expected trends in results, and did not allow for self-selection. We also know of no consequential changes in medical guidelines or technology in gynecological surgery, obstetrics or breast surgery at the time of program onset that would provide an alternative explanation for sudden, drastic improvements. Our approach of pooling many quality indicators and assuming trends for the average indicator can be considered a strength in this regard: When the indicator group is rich enough, it is unlikely that average trends are influenced by changes in clinical practice that affect a single or few indicators only. At the same time, the approach poses that it is possible and meaningful to average over indicators when estimating the DiD, without considering their clinical details. More fine-grained research questions might also require a more indicator-specific analysis.

While the causal relative risk estimate shows a large effect of the program, we have also presented an estimate of prevented adverse care events in absolute terms. Relative to the millions of patients whose medical procedures form part of the final analysis data set, the reported number may appear moderate. The reason is that clinical process deviations are in general a rare event – though with some variability across quality indicators (Table 1).

Is it possible to rule out unintended consequences of the policy? Previous research has discussed adverse effects of quality policies that create a strong pressure to perform [31, 37, 38]. Among those are the perverse incentive to preferably select low-risk patients, or to intentionally misreport. Both is no convincing concern for the program under study, as national-level case volume remained virtually unchanged (Supplementary information B), and data validation revealed that incorrect reporting was mostly to the own disadvantage. It would similarly be an unintended program effect if providers improved quality in medical procedures covered by program indicators at the expense of quality in other clinical areas (e.g., those captured by indicators in our control group). Note that this would also violate the *no interference* assumption (Supplementary information A), because the introduction of the program would have affected adverse event probability for patients treated in clinical areas not covered by the program. Yet, average quality did not deteriorate in the control group after program onset. Instead, it continued to slightly increase until the first COVID-19 year (Fig. 1A). A hypothesis of redirected rather than increased quality efforts is also not consistent with how authorities and hospitals reported to use program indicator results – with focus on broader quality measures and less on optimizing single metrics [30]. This would actually make *positive* spillovers of the policy across clinical departments in the same hospital more likely than negative. In such a case, the effect estimate in this study would be biased downward.

Importantly, to avoid confounding by trends in patient populations, this study excluded outcome quality indicators (2 out of 11 program indicators). Strictly speaking, conclusions hence only hold for quality of medical indications and hospital processes and do not speak to the question of patient outcomes. In general, process performance indicators might not strongly relate to health outcomes if they measure too narrow aspects of an institution’s care delivery [39].^8^ Still, all indicators in the program have been deemed important by expert groups for preventing severe patient hazards [7]. Theoretical frameworks typically view process quality as a causal precedent of patient outcomes, although the relationship should be scientifically established [42]. In that case, process indicators might even be preferable: “[When] they are relevant and practical, then they should be used in preference to outcome measures since they are much easier to interpret and are much more sensitive to differences in the quality of care” [20]. The sensitivity of process indicators to provider efforts could be a reason for the strong and rapid program effect we demonstrated. Effects of the program on patient outcomes might be more delayed, variable and/or smaller, but this remains speculation.^9^ With detailed data on patient characteristics, a risk-adjusted analysis of patient outcomes would be possible under the current DiD design, too.

Some caution in deriving general policy recommendations is necessary even when focusing only on process quality improvement. We estimated the average effect for the selected indicators (see definition of ATT in Supplementary information A), and did so in a very specific healthcare setting. It is not clear how well our results would generalize internationally, or even to other quality indicators and clinical areas. Healthcare systems and the institutional context of quality assurance vary widely across countries (examples are discussed in [9]). There are typically also no perfectly comparable quality indicators in terms of population and measured outcome. The latter prevents a comparison with international trends for the indicators analyzed in this paper.

Finally, it must be emphasized that our analysis merely concerns national-level results – the quality of care that can on average be expected by a German patient. This masks potential heterogeneity in quality of care and program effect between providers or regions. The lack of longitudinal provider-level results in the database also prevents any adjustment for provider effects or accounting for clustering (non-independence of observations from the same hospital), for example via multilevel models [44]. To alleviate this concern, in Supplementary information C we present the analysis of an alternative public data source that contains provider information but comes with other limitations. The results under adjustment for hospital effects confirm the main analysis.

## Conclusion

The program for the enhanced use of quality information for hospital capacity planning was effective in improving documented quality of care for German inpatients. This is of broad interest, since efforts towards more quality-based hospital capacity planning are also undertaken in other countries [45]. Our DiD analysis shows that a quality premium relative to existing statutory quality assurance was achieved when indicators were officially declared relevant for planning decisions. From a methodological perspective, we encourage to make more use of routinely collected data on quality indicators, which is often publicly available, for policy impact analysis. As we demonstrate, common quasi-experimental study designs can be adapted to these data and the study of quality policy effectiveness. Due to their often untestable assumptions, such analyses should complement more qualitative, in-depth evaluations that are already common in the field.

## Supplementary Information

Supplementary Material 1.

## Data Availability

Data are publicly available. The code to reproduce all analyses can be accessed at https://github.com/stefgehrig/qualityprogram.

## Abbreviations

G-BA: Federal Joint Committee (Gemeinsamer Bundesausschuss)
IQTIG: Institute for Quality Assurance and Transparency in Healthcare (Institut für Qualitätssicherung und Transparenz im Gesundheitswesen)
DiD: Difference-in-differences
ATT: Average treatment effect on the treated

## Footnotes

1 For 4 indicators in the year 2013 (IDs 52330, 52279, 50063, 2163), we replace initially published results by retrospective results published in the subsequent year, because they accommodate slight modifications in these indicators’ definitions that occurred from 2013 to 2014.

2 Of course, it is possible that common knowledge about the annual data validation component of the program incentivized higher documentation efforts in the program indicators. This would lead to better data quality due to the program even in the data *before* validation and recalculation.

3 Indicator definitions may vary from year to year and are thus published with a standardized assessment whether their results are “comparable”, “moderately comparable” or “not comparable” with results of the previous year.

4 One indicator was further removed manually in this step, because the event it measures is defined according to a percentile of the annual national-level distribution of the outcome. This prevents any between-year comparisons of event prevalence (ID 2133: Postoperative length of stay in liver transplantation).

5 The authors explored the effects of a policy change in mandatory quality reporting in the German healthcare system some years prior to our study period.

6 This is also supported by the alternative analysis presented in Supplementary information C. Results appear robust to adjusting for hospital effects.

7 Effects of the COVID-19 pandemic on quality indicators in community-acquired pneumonia in Germany have been analyzed in more detail in [35].

8 Previous studies in European healthcare settings on the association or process and outcome quality indicators have shown mixed results [40, 41]. They have also pointed out the difficulties in assessing such an association. These include weaknesses in risk adjustment of outcome indicators, lack of reliable data sources, or different levels of measurement.

9 In previous studies, outcome quality indicators in Germany showed less improvement over time compared to other indicator types [11, 43].
