# Supplementary material for: Estimating the causal effect of a quality assurance program on quality of care in Germany
Source: BMC Health Serv Res. 2025 Jun 9;25:815. doi: 10.1186/s12913-025-12939-8 (PMC12150581; doi:10.1186/s12913-025-12939-8)
Supplement: Supplementary file 1 — Supplementary Material 1. [file 12913_2025_12939_MOESM1_ESM.pdf]

Supplementary information for "Estimating the causal effect of a quality assurance program on quality of care in Germany"

Stefan Gehrig, Britta Zander-Jentsch, Maurilio Gutzeit, Silvia Klein, Johannes Rauh

Institute for Quality Assurance and Transparency in Healthcare (IQTIG), Katharina-Heinroth-Ufer 1, 10787 Berlin, Germany

Table of Contents

A Methodological details 2
A.1 Empirical strategy 2
A.2 Identifying assumptions 4
A.3 Estimation 6
B Additional figures 9
C Alternative data and analysis 12
Appendix references 14

A Methodological details

A.1 Empirical strategy

We are interested in the effect of the program on the risk of experiencing an adverse care event (the types of event are further described in section 2.1.2 of the main text) and among those patients for whom the program was in place, i.e., in the *treated* population. Accordingly, focus is on the patient population whose medical procedures (i) fall into quality indicators covered by the program and (ii) took place after the program was initiated with the start of the year 2017. We aim to compare the actually experienced risk in this population with the hypothetical risk they had experienced under the absence of the program. We express this comparison in terms of a marginal risk ratio.

Let Y denote the binary outcome that equals 1 if an adverse care event occurred for a patient and 0 otherwise. Likewise, the indicator D equals 1 if the program was in place for the quality indicator that covered a patient's medical procedure at the time of their healthcare visit and 0 otherwise. Hence, D denotes assignment status to the program. Superscripts, as in Y^D, are used to refer to potential outcomes under different counterfactual scenarios of program assignment (e.g., Hernán & Robins, 2020). The study population are all patients in Germany medically treated with procedures covered by any quality indicator in the final analysis data set (see section 2.1.2.3 of the main text) during the observation period. This period lasts from 2013 to 2020, i.e. from 4 years before until 4 years after program onset.

The quantity of interest then is

$$\tau_{ATT} = \frac{\mathbb{E}(Y^{D=1} \mid D = 1)}{\mathbb{E}(Y^{D=0} \mid D = 1)}. \qquad (1)$$

Here, τ_{ATT} describes a causal risk ratio for the *average treatment effect on the treated* (ATT; treatment here referring to the quality assurance program). In particular, it averages over the characteristics of the sub-population with $D = 1$, i.e., all patients affected by the program between 2017 and 2020. The goal of our statistical analysis will be to learn about τ_{ATT}, even though it cannot be estimated from data directly (see section A.2). If τ_{ATT} is below 1, the program was effective in reducing adverse care events.

The major challenge is to estimate in particular the unobserved, counterfactual quantity $\mathbb{E}(Y^{D=0} \mid D = 1)$ introduced above; that is, the expected proportion of adverse care events among those patients who were in the program, had they not been in the program. In the terminology of Lundberg et al. (2021), the *theoretical estimand* τ_{ATT} needs to be linked via identification assumptions to an *empirical estimand*, which we will call θ_{ATT}, that is estimable from empirical data. Our strategy is to apply the classic Difference-in-Differences (DiD) framework (Cunningham, 2021; Meyer, 1995). It is a widely used quasi-experimental method for impact evaluation of health policies (Basu et al., 2017; Craig et al., 2017; Wing et al., 2018). We adapt it to the binary outcome case, following previous work in economics (Blundell et al., 2004; Wooldridge, 2023). In spirit, the approach is similar to the "controlled before–after analysis" by Kraska et al. (2016). The authors explored the effects of a policy change in

mandatory quality reporting in the German healthcare system some years prior to our study period.

Since the program was introduced simultaneously for all hospitals in Germany, a DiD design with the same quality indicators in both hospitals within the program and in a control group is not possible (as in, e.g., the DiD study of performance-based incentives by Brenner et al., 2020). Instead, at the heart of our DiD approach is a comparison of changes in the proportion of adverse care events in a pre- and post-program period between indicators in the program group and a group of comparable quality indicators, for which the program was never introduced. The design can be understood as a repeated cross-section DiD: In each year, the population of patients undergoing a specific medical procedure is assessed, but we do not follow the same patients over the years.

The most critical assumption of DiD are *parallel counterfactual trends* (e.g., Cunningham, 2021, p. 414). It states that in the absence of the program, the results in the program group would have developed in parallel to the results in the control group. Put differently, the magnitude of all unobserved confounding remains constant over time and can hence be differenced out (Hernán & Robins, 2020, p. 97). The plausibility of this assumption for our study is discussed in section A.2. More precisely, we make the following assumption: Without the program, the program group average result would have developed in parallel to the average result in the control group. Since the outcome variable Y is binary and their expectation is bounded between 0 and 1, we assume the parallel trends on the logit scale.[1] The logit specification is a common choice for nonlinear DiD in binary or proportion outcomes (Blundell et al., 2004; Wooldridge, 2023).

The DiD, denoted by δ, is a group contrast of period contrasts:

$$\begin{aligned}\delta = \; & \Big(\text{logit}\big(\mathbb{E}(Y|t \geq 2017, q \in M)\big) - \text{logit}\big(\mathbb{E}(Y|t < 2017, q \in M)\big)\Big) - \\ & \Big(\text{logit}\big(\mathbb{E}(Y|t \geq 2017, q \notin M)\big) - \text{logit}\big(\mathbb{E}(Y|t < 2017, q \notin M)\big)\Big) \qquad (2)\end{aligned}$$

Here, we enumerate the 8 observed years by $t = 2013, \dots,\ 2020$, the 19 quality indicators by $q = 1, \dots,\ 19$ and indicate by $q \in M$ that the quality indicator q is part of the set M of quality indicators for which the program was introduced in 2017. This definition implies for the observed data that $D = 1 \Leftrightarrow q \in M \wedge t \geq 2017$.

The interpretation of δ is simple. For example, if a reduction in adverse care events from pre- to post-period was larger in the program group than in the control group (first row gives a smaller value than the second row), then δ would be negative.

We can now define the empirical estimand in terms of the risk ratio

$$\theta_{ATT} = \frac{\mathbb{E}(Y \mid D = 1)}{\text{logit}^{-1}\big(\text{logit}\big(\mathbb{E}(Y \mid D = 1)\big) - \delta\big)}. \qquad (3)$$

[1] In general, parallel trends are sensitive to nonlinear transformations of the mean, see Wooldridge (2023), especially when the difference in absolute outcome level is large between groups (Meyer, 1995).

Note that all terms on the right-hand side of equation (3) have simple estimators based on the observed data (see section A.3) and involve no potential outcomes. In the next section, we discuss assumptions, under which θ_{ATT} helps us to learn about τ_{ATT}.

A.2 Identifying assumptions

Under the assumptions laid out in this section - most importantly equation (4) - we get that $\tau_{ATT} = \theta_{ATT}$. In that case, we can interpret an estimate of θ_{ATT} as a estimate of the program effect on the treated population.

Arguing that θ_{ATT} is an (approximately) unbiased representation of the causal effect τ_{ATT} requires that the identifying assumptions are (approximately) met. The parallel counterfactual trends on the logit scale assumption can be expressed as

$$\begin{aligned}&\text{logit}\big(\mathbb{E}(Y^{D=0}|t \geq 2017, q \in M)\big) - \text{logit}\big(\mathbb{E}(Y^{D=0}|t < 2017, q \in M)\big) = \\ &\text{logit}\big(\mathbb{E}(Y^{D=0}|t \geq 2017, q \notin M)\big) - \text{logit}\big(\mathbb{E}(Y^{D=0}|t < 2017, q \notin M)\big). \qquad (4)\end{aligned}$$

It states that if there had never been a program, the difference in logit proportion of adverse care events between pre- and post-period would be the same for those quality indicators actually selected for the program (left-hand side of equation (4)) and those not selected (right-hand side of equation (4)). In short, results in the two groups would have followed the same time trends in the hypothetical absence of the program, even though their absolute levels of adverse care events might differ. Problems for the assumption are "time-by-group" interactions that are not due to the policy and not controlled by design or analysis (Zeldow & Hatfield, 2021).

There are at least five arguments in our setting in favor of the parallel trends assumption:

1. The selection of the 11 indicators for the program followed clear and well-documented criteria (IQTIG, 2016), see section 2.1.2 of the main text. Levels or, more importantly, current or expected future trends in results played no role for the decision-makers.

2. We chose quality indicators as a control group that are similar in important characteristics and constantly so over time (see section 2.1.2 of the main text). In fact, we reproduced parts of the original indicator selection process, but for other clinical areas. This increases the groups' general "comparability" (Meyer, 1995), making it more plausible that they would follow common trends.

3. The inspection of estimated pre-program trends (see Figure 1A of the main text) suggests that average indicator results move approximately in parallel between the two groups prior to the program. Trends start to diverge drastically only with the first program year 2017. Note that a small deviation from parallel pre-program trends already in the year 2016 (i.e., the last pre-intervention year) could be a minor announcement effect (Klein et al., 2023). Hospitals were informed about the selected program indicators already shortly before program implementation, although no program policies were put into place prior to January 2017.

4. The relative size of the indicator populations across repeated cross-sections, i.e., the amount of medical procedures conducted in each indicator, remains virtually unchanged over the whole observation period (Figure S2). Trends in population size would have been an indication that the composition of the underlying population might also change over time in an unaccounted way (as discussed by Cunningham, 2021, p. 460).

5. Two likely important potential confounders – quality indicator effects and year effects – are adjusted for during estimation (section A.3). For example, if the proportion of patients in quality indicators with generally lower frequency of adverse care events (i.e., events that can more easily be prevented; Table 1 of the main text shows the large heterogeneity) increased in the program group between pre- and post-program period, we could mistake this as an improvement in quality – though, in reality, it would just be a shift in healthcare system utilization by patients. Similar reasoning applies for year: There can be temporal variation in quality of care affecting all medical procedures – consider the first COVID-19 year 2020 – and the share of patients from "good" vs. "bad" years in the groups could differ between pre- and post-period. This is akin to the *conditional* parallel trends assumption (Wang et al., 2024, p. 631): parallel counterfactual trends only need to hold after controlling for the effect of indicator and year on the patient's probability to experience an adverse care event.

For θ_{ATT} to be a good representation of the causal effect τ_{ATT}, some additional assumptions must hold. There must be no effects of the program on quality experienced by patients prior to its introduction (*no anticipation*), see, e.g., Wooldridge (2023); Zeldow & Hatfield (2021):

$$Y^{D=0}|t < 2017 = Y^{D=1}|t < 2017$$

This is largely plausible since the final selection of quality indicators in the program was officially made public just shortly before becoming active in 2017 (G-BA, 2016). Any possible announcement effect (see above) should hence be minor, with the total pre-program observation we analyze spanning 4 years.

It is also assumed that the potential outcomes corresponding to actual program assignment have been observed (*consistency*). Indeed, the intervention we study is well-defined and was successfully turned into practice as intended for all selected quality indicators. It also applied uniformly across all treated patients in all German hospitals. Formally, this allows to replace the numerator in equation (1) by the numerator in equation (3).

Finally, implicit in our potential outcome notation (equation (1)), we assume potential outcomes of patients in one quality indicator to not depend on the program assignment status of any other quality indicator (*no interference*) – even if, for example, patients from that other quality indicator are treated in the same hospital. This assumption is largely plausible, as we did not include quality indicators in the control group from the same clinical areas as indicators in the program group. Therefore, control patients are treated in other hospital departments, and for other medical conditions. We briefly return to this question when discussing limitations in section 4.3 of the main text.

A.3 Estimation

The quality indicator result in year t for indicator q is just the mean of the outcomes, i.e., the fraction of adverse care events. Its logit transformation is denoted as α_{tq}. Let $i = 1, \ldots, n_{tq}$ denote the observed patients in a given quality indicator and year and the observed binary outcome of patient i in year t and indicator q as y_{tqi}. In a first step, we obtain maximum likelihood (ML) estimates of all quality indicator results on the logit scale via

$$\hat{\alpha}_{tq} = \text{logit}\left(\frac{1}{n_{tq}} \sum_{i=1}^{n_{tq}} y_{tqi}\right).$$

These estimates can equivalently be obtained by elementary operations on the observed variables as above, or from a logistic regression model. We use the regression model because it will later provide a convenient way to estimate standard errors. The model is parametrized with an intercept μ, simple effects for year and indicator, β_t and γ_q, respectively, and all indicator-year interaction effects ω_{tq}. To be identifiable, a reference category consisting of one combination of year and indicator is omitted:

$$\text{logit}\left(\mathbb{E}(y_{tqi})\right) = \mu + \beta_t + \gamma_q + \omega_{tq}, \text{ with } t \neq 2016 \text{ and } q \neq 1$$

The model has as many parameters as there are unique combinations of indicator and year in the full analysis data set ($19 \times 8 = 152$). The α_{tq} are linear combinations of those model parameters. For example, for the non-reference categories, we compute $\alpha_{tq} = \mu + \beta_t + \gamma_q + \omega_{tq}$.

Estimates $\hat{\alpha}_{tq}$ are then used to construct an estimate for δ. Specifically, the DiD of the logit-transformed mean outcomes in the 4 cells defined by period (pre and post) and group (program and control) is computed via

$$\begin{aligned}\hat{\delta}^w = \; & \left[\text{logit}\left(\frac{\sum_{t\geq 2017, q\in M} \text{logit}^{-1}\left(\hat{\alpha}_{tq}\right)}{\sum_{t\geq 2017, q\in M} 1}\right) - \text{logit}\left(\frac{\sum_{t<2017, q\in M} \text{logit}^{-1}\left(\hat{\alpha}_{tq}\right)}{\sum_{t<2017, q\in M} 1}\right)\right] - \\ & \left[\text{logit}\left(\frac{\sum_{t\geq 2017, q\notin M} \text{logit}^{-1}\left(\hat{\alpha}_{tq}\right)}{\sum_{t\geq 2017, q\notin M} 1}\right) - \text{logit}\left(\frac{\sum_{t<2017, q\notin M} \text{logit}^{-1}\left(\hat{\alpha}_{tq}\right)}{\sum_{t<2017, q\notin M} 1}\right)\right]. \qquad (5)\end{aligned}$$

The estimator is denoted with superscript w to indicate that we purposefully use a particular weighting of observations that does not follow directly from the definition of δ in equation (2). The "simple average of averages" in the calculation of $\hat{\delta}^w$ instead leads to each combination of t and q being weighted equally. This has two desirable consequences, we argue.

1. The analysis is adjusted for year and quality indicator effects. As laid out in section A.2, they could be confounders in the present DiD setting (Zeldow & Hatfield, 2021). The constant weighting ensures that the composition of patient populations from indicators with less and more prevalent adverse care events (i.e., less and more difficult to avoid departures from standard care processes, respectively) is held

constant across periods in both the program and control group. Also, the composition of patients from potentially “good” and “bad” years is held constant across groups in both the pre- and post-period.

2. It ensures that the DiD estimate is not dominated by those indicators with the largest patient populations (see Table 1 of the main text). The program was introduced on the quality indicator level. While throughout the analysis we assume that there is *a* program effect and not *multiple* program effects, and that assumptions hold for *all* patient populations, variability on the quality indicator level is a possibility.[2] For a recent overview on heterogeneity issues in DiD designs, see Wang et al. (2024). By weighting all indicator-specific mean outcomes equally, we gain some robustness against such variability. Note that, nevertheless, our estimator of the marginal risk ratio uses the observed distribution of patients across quality indicators and years (equation (3)). Using $\hat{\delta}^w$ simply implies that the mean outcome prediction under the counterfactual state of the world for each program quality indicator in the denominator of equation (3) relies on the estimated effect for an “average quality indicator”.

Recall that in equation (5), we average all $\hat{\alpha}_{tq}$ per group and *period* to yield the estimate of the DiD between pre- and post-program period on the logit scale. In an additional analysis, we average them per group and *year* to obtain estimates of annual pre- and post-trends of the two groups (again, under equal weighting of quality indicators). They are shown in Figure 1A of the main text. Contrasting these yearly group estimates in so-called event-study plots (see Figure 1B of the main text) makes it possible to gain insight into whether the two groups were comparable in their pre-trends, adjusted for potential trends in quality indicator composition, and into the dynamics of the program’s effect over time after its introduction (Cunningham, 2021, pp. 425–433).

Finally, we compute the marginal causal risk ratio estimate for the treatment effect on the treated

$$\hat{\theta}^w_{ATT} = \frac{\sum_{t \geq 2017, q \in M} n_{tq} \ \text{logit}^{-1}\left(\hat{\alpha}_{tq}\right)}{\sum_{t \geq 2017, q \in M} n_{tq} \ \text{logit}^{-1}\left(\hat{\alpha}_{tq} - \hat{\delta}^w\right)}, \qquad (6)$$

which averages over the observed distribution of quality indicators and years in the patient population affected by the program. The superscript w in $\hat{\theta}^w_{ATT}$ indicates that we plugged in $\hat{\delta}^w$ as DiD on the logit scale. Figure 1C of the main text shows results for the numerator and denominator of equation (6) separately.

[2] For example, in some medical procedures, program-induced improvements might materialize faster or more pronounced for unknown reasons. Also, general medical progress in a clinical area, coincident with program onset, might endanger the assumption of counterfactual parallel trends for some procedures, but not others.

To obtain estimates of standard errors and confidence intervals, we rely on the assumption that outcomes for a given year and quality indicator follow an i.i.d Binomial process.[3] The ML estimates $\hat{\alpha}_{tq}$ come with asymptotic standard errors based on the Fisher information (as implemented in standard logistic regression software packages). From there, we use the delta method to construct approximate 95% confidence intervals for all derived quantities.

All analyses were conducted in R v4.5.0 (R Core Team, 2025), using the library `marginaleffects` v0.25.1 for confidence interval estimation (Arel-Bundock et al., 2024). The analysis code is publicly available at https://github.com/stefgehrig/qualityprogram.

[3] This assumption is routinely used also by IQTIG when reporting uncertainty of national-level indicator results. However, we can expect that outcomes from patients treated by the same provider to be correlated. Without more fine-grained, provider-level data, we cannot account for this.

B Additional figures

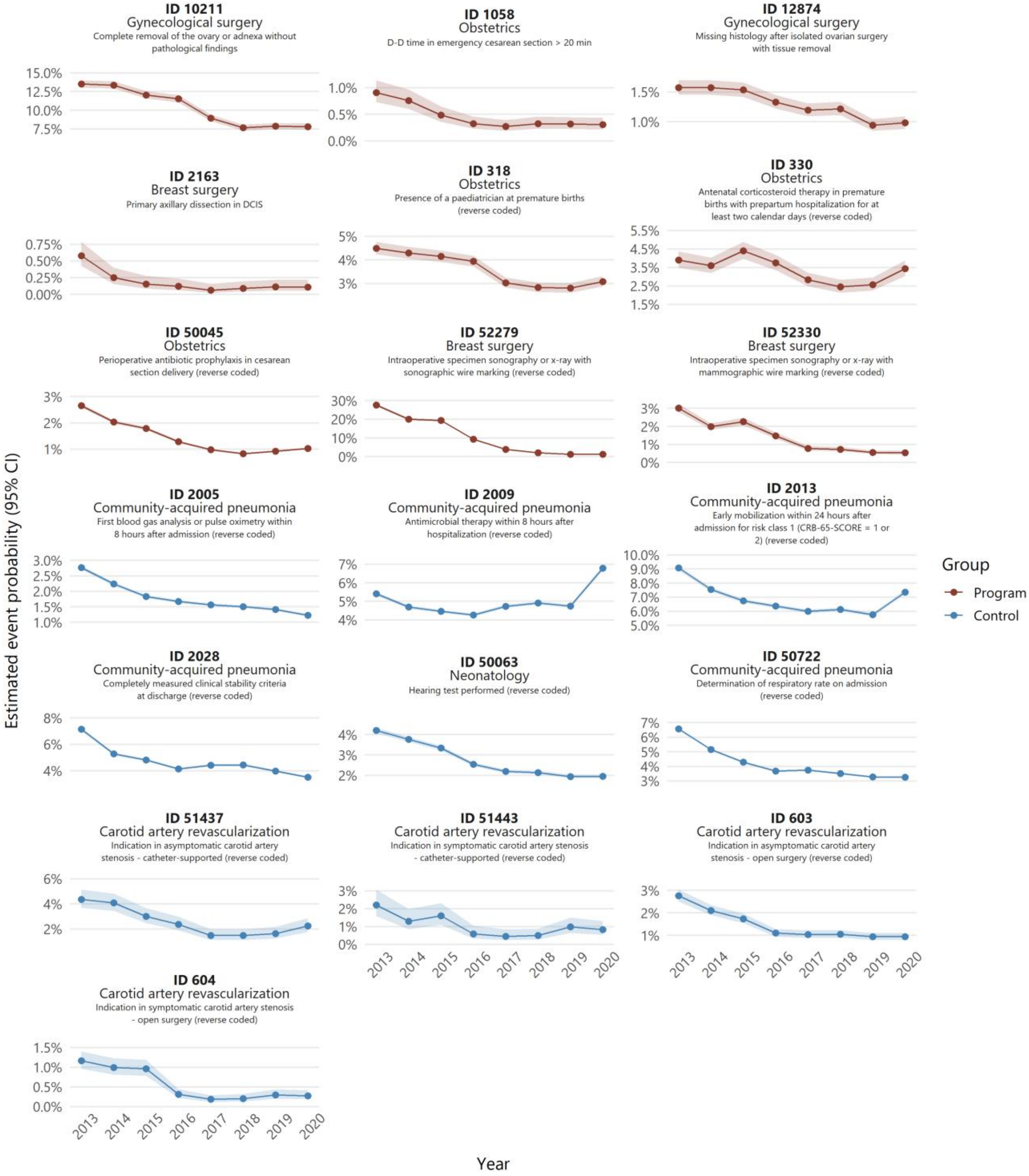

Figure S1: *Estimated time trends for each indicator with 95% Wilson confidence intervals.*

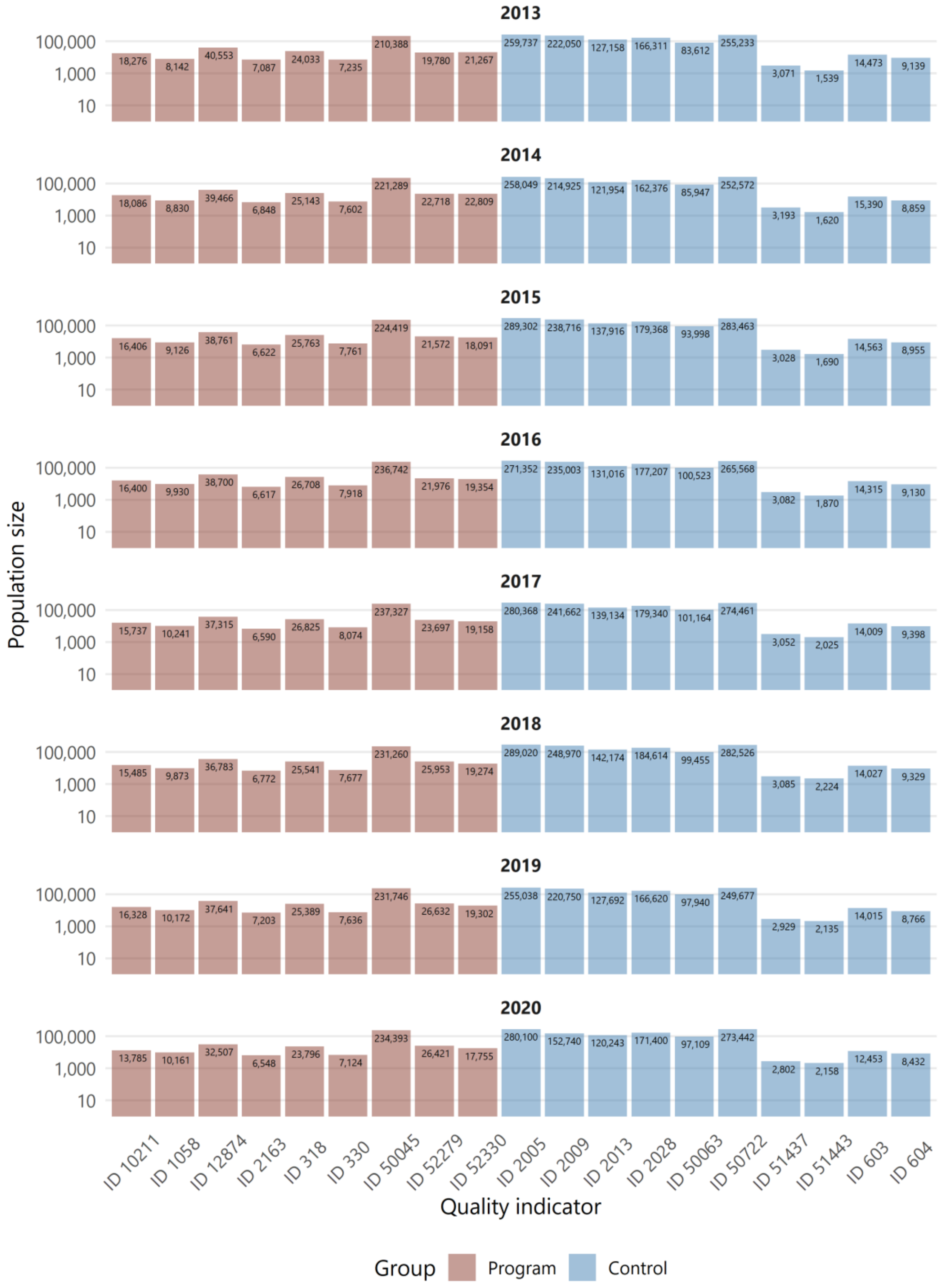

Figure S2: *Size of the population in each quality indicator (i.e., number of medical procedures in the indicator) for each year.*

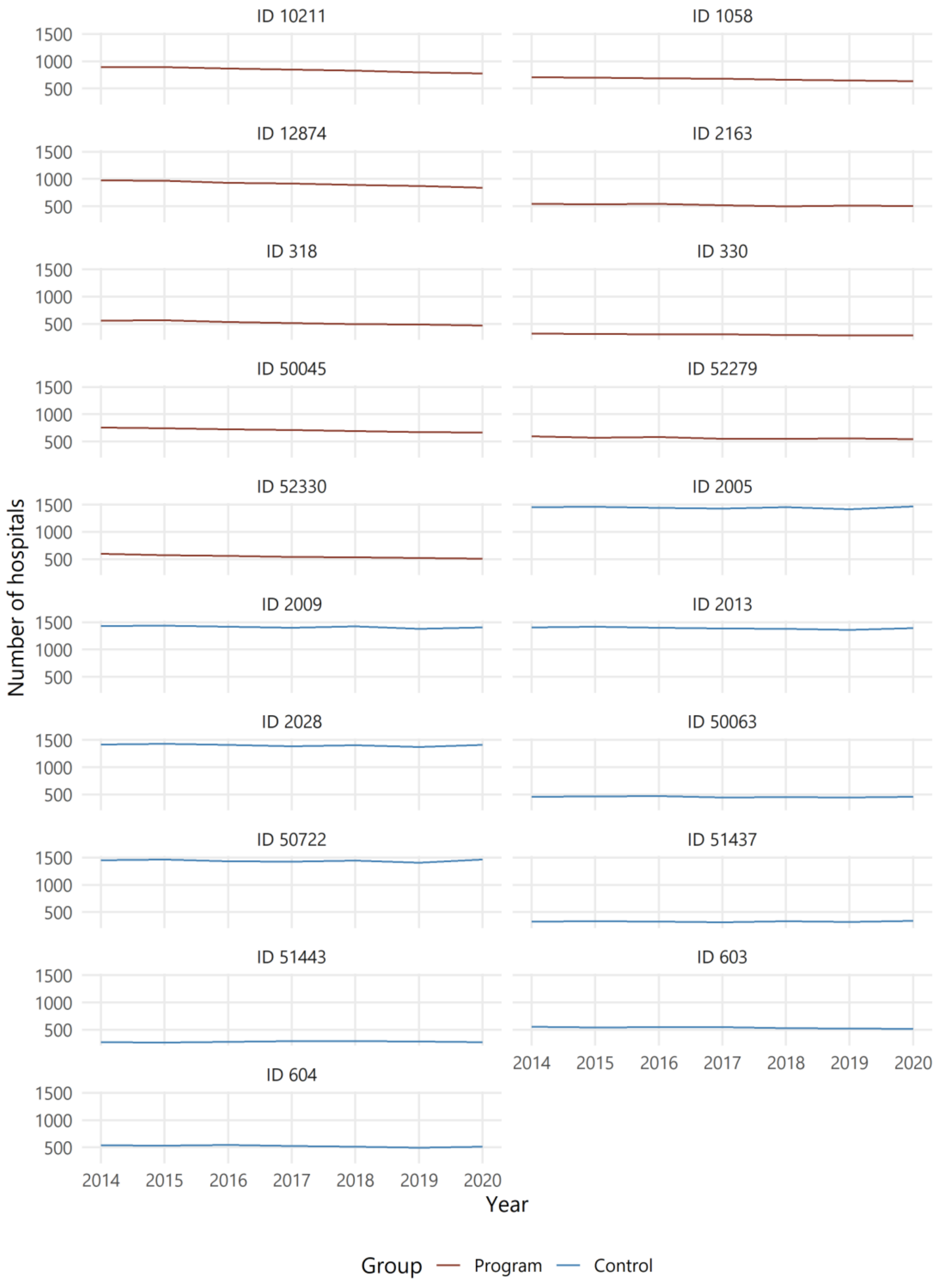

Figure S3: *Number of hospitals in each quality indicator for each year. There are no comparable data for 2013, because results were reported at a differently defined institutional level.*

C Alternative data and analysis

Hospitals in Germany are required to publish annual quality reports (*Qualitätsberichte der Krankenhäuser*) containing information on their structure (e.g., departments, personnel, medical procedures), patient volume, and quality. The reports contain results on some quality indicators, including those analyzed in this study. Data from the reports are collected and provided online by the Federal Joint Committee (*Gemeinsamer Bundesausschuss*) at https://qb-datenportal.g-ba.de/. Hence, the quality reports can be used as an alternative data source for the empirical analysis of our research question. In contrast to the database of annual national-level indicator results used in our main analysis, they contain hospital-level results. For another example for the use of these data, see Kraska et al. (2016).

The availability of hospital-level results allows for an analysis that accounts for heterogeneity in quality outcomes between individual hospitals. Specifically, we provide here an alternative analysis in which the logistic regression model used to obtain estimates $\hat{\alpha}_{tq}$, and ultimately $\hat{\delta}^{w}$ (see section A.3), includes normally distributed random hospital effects centered around zero (a mixed logistic regression model). Random provider effects are a suitable way to take into account the clustering of observations within healthcare providers (Ash et al., 2012; Gutzeit et al., 2025). As a consequence of the modeling approach, the resulting DiD estimate now needs to be interpreted conditionally, i.e., given a certain hospital quality level. In that sense, the DiD analysis of program impact is adjusted for hospital effects. By restricting the random effects structure to one random intercept per hospital, no indicator-specific hospital effects are assumed. Still, the approach leads to a model with 1,257 hospital effects (the distribution of estimates is shown in Figure S4A) and indicates considerably heterogeneity in hospital quality). The mixed logistic regression model is fit in `mgcv` (Wood, 2017). For presentation of the results in Figure S4B, we choose the average quality level by setting the random hospital effect to zero.

Hospitals' quality reports constitute a data source with well-known limitations (see also Kraska et al., 2016) and important disadvantages for the current research question:

- From the 8-year observation period in the current research design, 2 years need to be dropped (2013, 2014) because not all program indicators were included in hospital quality reports (they were not legally required for publication by hospitals).
- Due to data protection concerns, indicator results for a year and hospital were masked if the hospital's population in that indicator was below 4. This creates a vast amount of missing observations, which we drop.
- 2 of the 19 indicators in the current research design need to be dropped fully (IDs 1058, 2163), because in most years they do not exhibit any adverse care events in Germany anymore after deletion of masked results (see previous limitation). The resulting zero-probability strata would destabilize estimation of the logistic model.
- Hospital quality reports present results for program indicators *after* external data validation and correction. Since documentation errors discovered and corrected during data validation were mostly to the disadvantage of the provider (Klein et al.,

2023) and data validation was not applied for control group indicators, this could lead to inflated effect sizes estimates compared to the main analysis.

- The database is compiled by merging individually created reports from different years and providers, if available. The resulting data quality is imperfect. We correct obvious errors or duplicates, but a verification, completeness check or plausibility check of all individual results is infeasible. It is possible that reports for some hospitals and years are missing in the database (see also Kraska et al., 2016).

- Reliable matching of hospitals across years was only possible in terms of the overarching institution (*Institutionskennzeichen*), which is not the provider level at which quality data in Germany is usually collected and analyzed. Rather, statutory quality assurance has been applied to units at the more fine-grained level of individual sites (*Standorte*) since the year 2014. Different sites of the same institution might deliver different levels of quality of care. In that sense, the meaning of the term "hospital" differs in the results in Figure S3 (sites) and in Figure S4 (institutions).

Due to the above limitations together with the different interpretation (conditional rather than marginal DiD), we discourage a direct comparison with the main results in quantitative terms, as well as a too literal reading of effect size. Nevertheless, we understand the alternative data and analysis presented here as a valuable robustness check. Figure S4B shows an event plot of the same type as Figure 1B of the main text and suggests a strong impact of the program on process quality. An assessment of parallel pre-trends is difficult due to the lack of years 2013 and 2014.

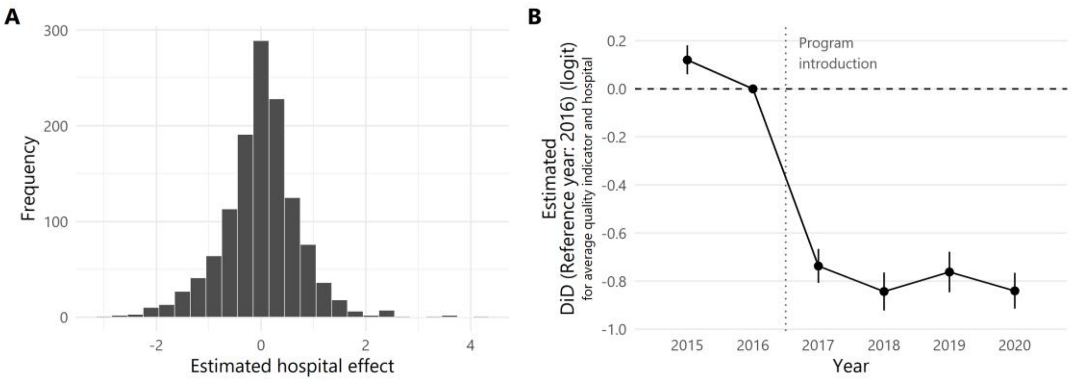

Figure S4: *(A) Frequency distribution of estimated random hospital effects from the mixed logistic regression model (estimated population standard deviation: 0.78). (B) Events study plot showing DiD estimates for every year relative to the difference between groups in the year 2016, with 95% confidence intervals. The plot is based on the analysis of data from hospital quality reports.*

Appendix references

Arel-Bundock, V., Greifer, N., & Heiss, A. (2024). How to Interpret Statistical Models Using marginaleffects for R and Python. *Journal of Statistical Software*, *111*(9), 1–32. https://doi.org/10.18637/jss.v111.i09

Ash, A. S., Fienberg, S. E., Louis, T. A., Normand, S.-L. T., Stukel, T. A., & Utts, J. (2012). *Statistical Issues in Assessing Hospital Performance*. Committee of Presidents of Statistical Societies (COPSS). https://www.cms.gov/Medicare/Quality-Initiatives-Patient-Assessment-Instruments/HospitalQualityInits/Downloads/Statistical-Issues-in-Assessing-Hospital-Performance.pdf

Basu, S., Meghani, A., & Siddiqi, A. (2017). Evaluating the Health Impact of Large-Scale Public Policy Changes: Classical and Novel Approaches. *Annual Review of Public Health*, *38*(1), 351–370. https://doi.org/10.1146/annurev-publhealth-031816-044208

Blundell, R., Meghir, C., Dias, M. C., & Reenen, J. V. (2004). Evaluating the Employment Impact of a Mandatory Job Search Program. *Journal of the European Economic Association*, *2*(4), 569–606. https://www.jstor.org/stable/40004874

Brenner, S., Chase, R. P., McMahon, S. A., Lohmann, J., Makwero, C. J., Muula, A. S., & De Allegri, M. (2020). Effect Heterogeneity in Responding to Performance-Based Incentives: A Quasi-Experimental Comparison of Impacts on Health Service Indicators Between Hospitals and Health Centers in Malawi. *Health Systems & Reform*, *6*(1), e1745580. https://doi.org/10.1080/23288604.2020.1745580

Craig, P., Katikireddi, S. V., Leyland, A., & Popham, F. (2017). Natural Experiments: An Overview of Methods, Approaches, and Contributions to Public Health Intervention Research. *Annual Review of Public Health*, *38*(1), 39–56. https://doi.org/10.1146/annurev-publhealth-031816-044327

Cunningham, S. (2021). *Causal Inference: The Mixtape*. Yale University Press.

G-BA. (2016). *Beschluss des Gemeinsamen Bundesausschusses über die Liste der Qualitätsindikatoren gemäß § 136c Absatz 1 des Fünften Buches Sozialgesetzbuch (SGB V): Liste planungsrelevanter Qualitätsindikatoren*. https://www.g-ba.de/downloads/39-261-2816/2016-12-15_PlanQI-RL_Liste-planQI_BAnz.pdf

Gutzeit, M., Rauh, J., Kähler, M., & Cederbaum, J. (2025). Modelling Volume-Outcome Relationships in Health Care. *Statistics in Medicine*, *44*(6), e10339. https://doi.org/10.1002/sim.10339

Hernán, M. A., & Robins, J. M. (2020). *Causal Inference: What If*. Chapman & Hall/CRC.

IQTIG. (2016). *Planungsrelevante Qualitätsindikatoren. Abschlussbericht zur Auswahl und Umsetzung*. https://iqtig.org/dateien/berichte/2016/IQTIG_Planungsrelevante-Qualitaetsindikatoren_Abschlussbericht.pdf

Klein, S., Rauh, J., Pauletzki, J., Klakow-Franck, R., & Zander-Jentsch, B. (2023). Introduction of quality indicators in German hospital capacity planning – Do results show an improvement in quality? *Health Policy*, *133*, 104830. https://doi.org/10.1016/j.healthpol.2023.104830

Kraska, R. A., Krummenauer, F., & Geraedts, M. (2016). Impact of public reporting on the quality of hospital care in Germany: A controlled before–after analysis based on secondary data. *Health Policy*, *120*(7), 770–779. https://doi.org/10.1016/j.healthpol.2016.04.020

Lundberg, I., Johnson, R., & Stewart, B. M. (2021). What Is Your Estimand? Defining the Target Quantity Connects Statistical Evidence to Theory. *American Sociological Review*, *86*(3), 532–565. https://doi.org/10.1177/00031224211004187

Meyer, B. D. (1995). Natural and Quasi-Experiments in Economics. *Journal of Business & Economic Statistics*, *13*(2), 151–161. https://doi.org/10.2307/1392369

R Core Team. (2025). *R: A Language and Environment for Statistical Computing*. R Foundation for Statistical Computing. https://www.R-project.org/

Wang, G., Hamad, R., & White, J. S. (2024). Advances in Difference-in-differences Methods for Policy Evaluation Research. *Epidemiology*, *35*(5), 628. https://doi.org/10.1097/EDE.0000000000001755

Wing, C., Simon, K., & Bello-Gomez, R. A. (2018). Designing Difference in Difference Studies: Best Practices for Public Health Policy Research. *Annual Review of Public Health*, *39*(1), 453–469. https://doi.org/10.1146/annurev-publhealth-040617-013507

Wood, S. N. (2017). *Generalized Additive Models: An Introduction with R* (2nd ed.). Chapman; Hall/CRC.

Wooldridge, J. M. (2023). Simple approaches to nonlinear difference-in-differences with panel data. *The Econometrics Journal*, *26*(3), C31–C66. https://doi.org/10.1093/ectj/utad016

Zeldow, B., & Hatfield, L. A. (2021). Confounding and regression adjustment in difference-in-differences studies. *Health Services Research*, *56*(5), 932–941. https://doi.org/10.1111/1475-6773.13666
